# COVID-19-Induced Thrombosis in Patients without Gastrointestinal Symptoms and Elevated Fecal Calprotectin: Hypothesis Regarding Mechanism of Intestinal Damage Associated with COVID-19

**DOI:** 10.3390/tropicalmed5030147

**Published:** 2020-09-16

**Authors:** Mauro Giuffrè, Stefano Di Bella, Gianluca Sambataro, Verena Zerbato, Marco Cavallaro, Alessandro Agostino Occhipinti, Andrea Palermo, Anna Crescenzi, Fabio Monica, Roberto Luzzati, Lory Saveria Crocè

**Affiliations:** 1 Department of Medical, Surgical and Health Sciences, University of Trieste, 34151 Trieste, Italy; stefano932@gmail.com (S.D.B.); roberto.luzzati@asuits.sanita.fvg.it (R.L.); lcroce@units.it (L.S.C.); 2 Italian Liver Foundation, 34149 Basovizza, Trieste, Italy; 3 University Hospital “Policlinico-Vittorio Emanuele”, Department of Clinical and Experimental Medicine, University of Catania, 95100 Catania, Italy; dottorsambataro@gmail.com; 4 Infectious Diseases Department, Udine University, 33100 Udine, Italy; verena.zerbato@gmail.com; 5 Department of Radiology, Trieste University Hospital, 34139 Trieste, Italy; mrc.cavallaro@virgilio.it; 6 Emergency Department, Trieste University Hospital, 34139 Trieste, Italy; alexander82@tiscali.it; 7 Unit of Endocrinology and Diabetes, Campus Bio-Medico University, 00128 Rome, Italy; a.palermo@unicampus.it; 8 Section of Pathology, Campus Bio-Medico University, 00128 Rome, Italy; a.crescenzi@unicampus.it; 9 Department of Gastroenterology, Trieste University Hospital, 34139 Trieste, Italy; fabio.monica@asuits.sanita.fvg.it

**Keywords:** fecal calprotectin, COVID-19, SARS-CoV-2, bowel perforation, D-Dimer, thrombosis, ischemia

## Abstract

Background: Patients with coronavirus infectious disease 2019 (COVID-19) and gastrointestinal symptoms showed increased values of fecal calprotectin (FC). Additionally, bowel abnormalities were a common finding during abdominal imaging of individuals with COVID-19 despite being asymptomatic. The current pilot study aims at evaluating FC concentrations in patients without gastrointestinal symptoms. Methods: we enrolled 25 consecutive inpatients with COVID-19 pneumonia, who were admitted without gastrointestinal symptoms and a previous history of inflammatory bowel disease. Results: At admission, 21 patients showed increased FC with median values of 116 (87.5; 243.5) mg/kg despite absent gastrointestinal symptoms. We found a strong positive correlation between FC and D-Dimer (r = 0.745, *p* < 0.0001). Two patients developed bowel perforation. Conclusion: our findings may change the current understanding of COVID-19 intestinal-related disease pathogenesis, shedding new light on the potential role of thrombosis and the consequent hypoxic intestinal damage.

## 1. Main Text

At the time of writing this letter, the principal cause of mortality in patients with severe acute respiratory syndrome coronavirus 2 (SARS-CoV-2) infection is respiratory failure with exudative diffuse alveolar damage and massive capillary congestion often accompanied by microthrombi or, in lower percentages, by generalized thrombotic microangiopathy, as reported by post-mortem examinations [1]. With coronavirus infectious disease 2019 (COVID-19) being conceived as a solely respiratory disease [2,3], the scientific community initially assumed that the lungs represented the preferred and initial site of viral proliferation, as well as its primary source for shedding and transmission.

## 2. Gastrointestinal Manifestations of COVID-19

Recent findings indicated that SARS-CoV-2 could bind to gastrointestinal cells via specific receptors (such as the angiotensin converting enzyme-2 (ACE2) receptor and the transmembrane serine protease 2) [4], whose interaction is supposed to promote local inflammation by massive cytokine and chemokine release [5]. Additionally, an autoptic study on the small intestine of two COVID-19 patients showed endotheliitis of the submucosa vessels and evidence of direct viral infection of endothelial cells [6].

Regarding the clinical presentation, gastrointestinal symptoms are present in up to 28% of patients with COVID-19 [7,8,9], and fecal SARS-CoV-2-RNA was detected in approximately 50% of positive individuals [8,9,10]. Recently, Effenberger et al. [5] proposed the role of fecal calprotectin (FC) as a marker of intestinal inflammation in COVID-19 patients who developed gastrointestinal (GI) symptoms. In particular, the authors detected significantly higher FC values in patients with acute diarrhea if compared to patients without diarrhea or with ceased diarrhea (>48 h). In this pilot study, we aimed to externally validate the results of Effenberger et al. [5] by determining the actual absence of significant FC concentrations in patients who did not present gastrointestinal symptoms and without previous history of inflammatory bowel disease (IBD).

## 3. Materials and Methods

We performed a prospective observational cross-sectional study by enrolling 25 consecutive COVID-19 inpatients from May 2020 to June 2020, who were admitted without gastrointestinal symptoms and a previous history of IBD.

SARS-CoV-2 detection in nasopharyngeal swabs was determined by PCR (LightMix^®^-Modular-Wuhan CoV-RdRP and E genes). FC concentration was determined by the DiaSorin-LIAISON^®^-Calprotectin according to the manufacturer’s specification (normal range <50 mg/kg). Both tests were performed at admission. Potential infective causes for FC increase were investigated through canonical stool analysis for common bacteria, viral and parasitic pathogens.

Data were displayed as median (Quartile 1; Quartile 3). We explored the correlation between continuous variables via Spearman’s correlation coefficient, considering a statistically significant two-tailed *p*-value < 0.05.

## 4. Results

From the 25 original patients, 21 (84%) showed increased FC, with median values of 116 (87.5; 243.5) mg/kg despite being asymptomatic for GI symptoms, and a median D-Dimer of 1.32 (0.82; 2) mg/L (normal value < 0.5 mg/L). Their clinical characteristics, possible risk factors, and biochemical parameters are reported in Table 1. We did not detect any significant correlation between FC and the C reactive protein (CRP), white blood cell count (WBC), platelet count, ferritin, lactate dehydrogenase (LDH) or albumin. However, we found a strong positive correlation between FC and D-Dimer (r = 0.745, *p* < 0.0001).

### 4.1. Intestinal Perforation

Two of the enrolled patients developed intestinal perforation. They were admitted to the emergency department for high-grade fever, cough and dyspnea. Both resulted positive to SARS-CoV-2 in nasopharyngeal swabs, and according to their comorbidities, they were admitted to the hospital.

The first patient, a 68-year-old female with an FC of 216 mg/kg, WBC of 17,940 cells/mm^3^, CRP of 94 mg/L and D-Dimer of 1.28 mg/LFEU developed severe abdominal pain 20 days after hospital admission. The contrast-enhanced abdominal computed tomography (CT) showed rectal wall and sigmoid colon thickening surrounded by extraluminal free air; a small-sized fluid collection and a smoothly thickened peritoneum were noted. The patient was treated conservatively.

The second patient, an 84-year-old female with an FC of 290 mg/kg, WBC of 5990 cells/mm^3^, CRP of 290 mg/dL and D-Dimer of 2.1 mg/LFEU developed severe abdominal pain the day after admission and septic shock. The contrast-enhanced abdominal CT showed rectal wall thickening, with free air organized circumferentially around the mesorectum, suggestive for retroperitoneal perforation; an inflammatory stranding of perivisceral fat tissue was also identified. The patient died the next day.

### 4.2. A Hypothesis on the Role of Thrombosis

In contrast to Effenberger et al. [5], we detected an increase in FC concentration in 88.2% of patients without GI symptoms. Besides, our findings also represent the first report of a significant positive correlation between FC and D-Dimer. This particular discovery may change our current understanding of COVID-19 intestinal-disease pathogenesis, shedding new light on the potential role of thrombosis and the consequent hypoxic intestinal damage. Indeed, FC is mainly expressed by neutrophils [11], whose functions are severely affected by intestinal ischemia [12]. The two patients that developed bowel perforation showed on the CT rectal and sigmoid colon wall thickening. In non-COVID-19 patients, left flexure and sigmoid colon segments have the highest risk of ischemic colitis, while distal rectum is usually spared due to its dual blood supply; at variance, in COVID-19 patients colon/rectal thickening was previously reported in seven cases [13] in agreement with our data, supporting a relationship between intestinal damage and COVID-19 infection. Notably, D-Dimer was found to increase in up to 47% of patients with COVID-19 at hospital admission [14], which resulted in higher mortality rates despite not presenting pulmonary embolism or deep vein thrombosis. Was the gut being responsible for these patients’ death? Unfortunately, this question has no definitive answer, but according to a recent study, bowel abnormalities were a common finding (31%) during abdominal imaging of individuals with COVID-19. Patients who underwent laparotomy often showed histological ischemia due to small vessel thrombosis [13].

That being said, our data should be taken with caution due to the relatively small sample size and the possible limitations related to selection bias. While waiting for the additional validation, and data on gut histopathology (autoptic reports or in vivo endoscopic biopsies) in COVID-19 patients, we firmly believe that the correlation between FC and D-Dimer may yield the secrets behind the COVID-19 Pandora’s box.

## Figures and Tables

**Table 1:** Patients’ Characteristics and Laboratory Parameters. Continuous Variables are Reported as the Median (Quartile1; Quartile3). CRP: C Reactive Protein; LDH: Lactate Dehydrogenase.

Characteristics	Patients with Abnormal Fecal Calprotectin *N* = 21	Patients without Abnormal Calprotectin *N* = 4
Age, years	78.3 (69; 81.5)	76 (70; 84)
Gender, Male	15 (71.4%)	3 (75%)
BMI (kg/m^2^)	24 (21; 27)	25 (20; 26)
Hearth Diseases	10 (47.6%)	2 (50%)
Hypertension	7 (33.3%)	2 (50%)
Previous ACE-Inhibitors	5 (23.8%)	2 (50%)
Active Smokers	7 (33.3%)	1 (25%)
Antibiotic Therapy	5 (23.8%)	1 (25%)
Heparin Therapy	13 (61.9%)	2 (50%)
White Blood Cell Count	6530 (4050; 7350)	6110 (4350; 8365)
Platelets (10^9^/L)	205 (141;357)	189 (153; 265)
PCR (mg/dL)	31 (24; 91)	40 (8; 115)
LDH (U/L)	290 (195; 381)	250 (190; 300)
Ferritin (µg/L)	645 (520; 1210)	550 (420; 610)
Fecal Calprotectin (mg/kg)	116 (87.5; 243.5)	
D-Dimer (mg/LFEU)	1.32 (0.82; 2)	0.87 (0.5; 1.23)
